# Ecotype-Level Genetic Biodiversity of Five Italian Traditional Crops

**DOI:** 10.1155/2019/4652769

**Published:** 2019-07-01

**Authors:** Francesco Guarino, Stefano Castiglione, Giovanni Improta, Maria Triassi, Angela Cicatelli

**Affiliations:** ^1^Dipartimento di Chimica e Biologia “Adolfo Zambelli”, Università degli Studi di Salerno, Via Giovanni Paolo II, 132, 84084 Fisciano (SA), Italy; ^2^Dipartimento di Sanità Pubblica, Università degli Studi di Napoli Federico II, Via Pansini, 5, 80125 Napoli (NA), Italy

## Abstract

Italy displays a high level of agrobiodiversity due to its diversified pedoclimatic zones. The Administrative Region of Campania includes several and divergent biomes, occurring close to each other. In fact, the distance between a sea level environment and that of high mountains can be less than 20 km. These environmental conditions allow the cultivation of many different crops and vegetables, represented by diverse ecotypes and varieties that are well adapted to the distribution range where they have been selected and grown. Efforts to maintain and further increase biodiversity in farming systems require a better understanding of the existing diversity created by traditional farming practices. The aim of our study was to identify and molecularly characterize several ecotypes belonging to five horticultural species commonly cultivated in Campania. In particular, we analysed five ecotypes of maize, two of garlic, four of onion, one of escarole, and two of courgette by means of simple sequence repeat (SSR) markers in order to evaluate their level of genetic biodiversity. The results reveal, for the first time, the high genetic biodiversity of horticultural ecotypes of the Campania Region. This feature is very important to improve the quality and productivity of agroecosystems.

## 1. Introduction

In agroecosystems, genetic diversity is widely regarded as pivotal in sustaining and preserving ecosystem functioning and services [1]. Traditional crops, in particular, constitute the largest reservoir of genetic diversity, and apart from their importance from an ecological point of view, their conservation has great social, economical, and cultural implications [2, 3]. Indeed, traditional crops, and especially landraces, have a history of cultivation and adaptation to local environmental conditions, which translates into unique tastes or nutritional properties as well as higher resistances to biotic and abiotic stresses [4]. From the “Green Revolution” onwards, however, humans focused their attention only on some species that were considered the most interesting, and within these species, they exerted a selective pressure toward specific phenotypes, thus causing a reduction in allelic diversity. The consequence has, therefore, been the replacement of species and/or traditional and local varieties by highly selected and specialised ones, leading to an overall loss in biodiversity [5] since only a subset of the diversity of the wild species remains after repeated selection for desired traits.

Crop biodiversity has been influenced by a number of genetic bottlenecks occurring during domestication and through modern plant breeding [6]. Nevertheless, existing plant genetic resources can still provide a biological and fundamental basis for world food security and support the livelihoods of human populations. These resources are regarded by breeders and farmers as the most important raw material. Thus, traditional agriculture and local knowledge of agricultural biodiversity, cultural factors, and participatory processes, as well as tourism associated with agricultural landscapes, are the new challenges for sustainable development [7]. Many traditional crops and landraces survive primarily, thanks to local farmers, who are the keepers of an invaluable biodiversity heritage. Although, in general, the greatest loss of diversity occurred from the landraces than the wild plants, the older farmers, in particular, have become the principal depositories of traditional knowledge, mostly coming from family traditions and handed down through the generations [8]. They have identified and propagated by means of simple selection procedures and specific landraces deemed of particular interest. In this case, domestication causes a net increase in diversity [9]. In fact, behind the rediscovered interest in traditional crops and landraces, there is also the recognition of their important role as sources of genetic diversity, which is potentially useful for crop improvement and sustainable agriculture. Today, biodiversity meets and stimulates the various requirements of the market (in terms of quality standard, rediscovery of traditions, etc.) of the production sector (in terms of plants more tolerant to climate change, new cultivation methods, or biotic stresses) as well as the needs of the processing industry and of modern distribution.

Italy, and in particular southern Italy, displays a broad crop biodiversity, related to its heterogeneity at both the landscape and social/cultural level [10]. However, the current conservation landscape does not reflect the Mediterranean biome's rarity and its importance for plant endemism [11]. Habitat conversion will clearly outpace the expansion of formal protected-area networks, and conservationists will have to replace traditional strategies with new approaches to sustain the Mediterranean biota. Using regional-scale datasets, it is possible to determine the area of land that is protected, converted (*e.g.*, urban or industrial), impacted (*e.g.*, intensive and cultivated agriculture), or lands that can be subjected to conservation potential. The natural and seminatural lands that are unprotected (*e.g.*, private range lands) can sustain numerous native species and associated habitats.

Campania stands out for its extreme landscape heterogeneity and exceptional geomorphological and pedogenetic traits. Thanks to its highly fertile soils, it was called “Campania Felix” during the Roman Empire and crops cultivated on the volcanic soils near Vesuvio were regarded by Goethe as being unparalleled [12]. Landscape heterogeneity is reflected in the presence of numerous ecotypes, which are historically adapted to the areas where they are grown.

In order to protect its local horticultural ecotypes, the Administrative Region of Campania has launched a project called “Agrigenet,” a network for the protection and management of agrofood genetic resources. The project includes a biodiversity study of the regional ecotypes and their conservation both in situ, by keeper farmers, and ex situ through the establishment of a germplasm bank.

Several studies have been conducted to evaluate the biodiversity in horticultural products in different countries and regions of the world [13–16], but much of the existing biodiversity is still unknown. Our study was aimed at genetically characterizing several ecotypes belonging to five horticultural species from the Campania Region, in order to estimate their biodiversity and contribute to their conservation in the framework of the Agrigenet project. For this purpose, five ecotypes of maize (*Zea mays* L.), four of onion (*Allium cepa* L.), two of garlic (*Allium sativum* L.), one of escarole (*Cichorium endivia* L.), and two ecotypes of courgette (*Cucurbita pepo* L.) were molecularly characterized using a set of highly polymorphic microsatellite (Simple Sequence Repeat, SSR) markers.

## 2. Materials and Methods

### 2.1. Plant Material

The different accessions of diverse ecotypes of maize, onion, garlic, escarole, and courgette were provided by the Agricultural Research Council (CRA-SCS, Battipaglia, SA, Italy) and by the National Research Institute for Food and Nutrition (INRAN, Pontecagnano, SA, Italy). They were selected on the basis of the farmers' local knowledge. In origin, the institution obtained the ecotypes from the keeper farmers, but no information is available regarding the contribution of the farmers to these collections. The details of the furnished accessions are given in Table 1.

### 2.2. SSR Analysis

Leaf samples for DNA isolation were collected from seedlings of each accession. Total DNA was extracted from leaves using DNeasy Plant Mini Kit (Qiagen, Milano, Italia). Quality and quantity were assessed by DNA electrophoresis in 1.0% agarose gel with reference standards and by evaluating the 260/280 ratio with a Picodrop Microliter UV/Vis Spectrophotometer (Picodrop, UK). A total of 35 SSR primer pairs (seven for maize, six for garlic, seven for onion, eight for escarole, and seven for courgette) were used for PCR amplifications. The primer sequences are given in Table SM1. PCR reactions were performed in a volume of 10 *μ*l containing 20 ng DNA, 1.2–4.0 pmol of each primer, 10x PCR buffer, 1.25 mM dNTPs, and 0.5 units of *Taq* polymerase. Amplifications were performed using the following PCR amplification conditions: 94°C for 2 minutes followed by 30 cycles of 94°C for 30 seconds, X°C for 1 minute (X indicates the annealing temperature reported in Table SM1), and 72°C for 1 minute followed by final extension at 72°C for 5 minutes. PCR products were separated on ABI Prism 310 Genetic Analyzer (Applied Biosystems, Milano, IT), adding 500 ROX (Applied Biosystems, Milano, IT) as internal size standard. SSR fragments were analysed using Gene Mapper V 3.7. The SSR fragments were finally scored as length of the electrophoresed alleles for each investigated locus.

### 2.3. Data Analysis

Polymorphism Information Content (PIC) values or expected heterozygosity scores for SSR (polyallelic) markers were calculated using the formula: Hj = 1 − ∑pi2, where pi is the frequency for the *i*th allele [17].

The most used and informative indices for population genetics studies are the observed and expected heterozygosity. It can also refer to the fraction of loci within individuals that are heterozygous. Typically, the observed (*H*_o_) and expected (*H*_e_) heterozygosity are considered.

*F*_ST_, *F*′, *Φ*_ST_, *Φ*′_ST_, and *D*_est_ are the primary indices used for estimating and testing the level of genetic divergence among populations. *F*_ST_ is the best choice for datasets consisting of neutral polymorphism datasets involving two alleles per locus. *D*_est_ and *F*′ tend to be the suitable index for analyses where more than two alleles are considered.

The Shannon Index (I) was calculated on a single-locus basis, where ln is the natural logarithm and *p*_*i*_ is the frequency of the *i*th allele. Unlike *H*_e_, *I* is not bounded by 1 and may, therefore, be a better measure of allelic and genetic diversity, though largely overlooked in genetic studies:

(1) $$\begin{array}{c} \text{I}=\sum p_{\text{i}}\ln\;p_{\text{i}}. \end{array}$$

The genetic similarity matrices of all accessions were elaborated using Jaccard's similarity coefficient (JSC) [18]. A cluster analysis was constructed on the similarity matrix by means of the unweighted pair group mean with arithmetical averages (UPGMA) method using NTSYSpc software (Numerical Taxonomy System, version 2.1) based on the JSC.

Structure version 2.2 software [19] was used to define the optimal number of clusters and to infer the population structure using the genetic profiles of each ecotype. The number of populations (K) was estimated by performing 10 runs for each population, from *K* = 1 to *K* = 10. Each run consisted of 100,000 MCMC (Markov Chain Monte Carlo) permutations with a burn-period of 10,000, assuming no *a priori* information on population affiliation, the admixture, and correlated allele frequencies methods. The optimal population structure was estimated using the method of Evanno et al. [20] with 20 independent runs for each *K* value.

## 3. Results

### 3.1. Maize

**Figure 1:**
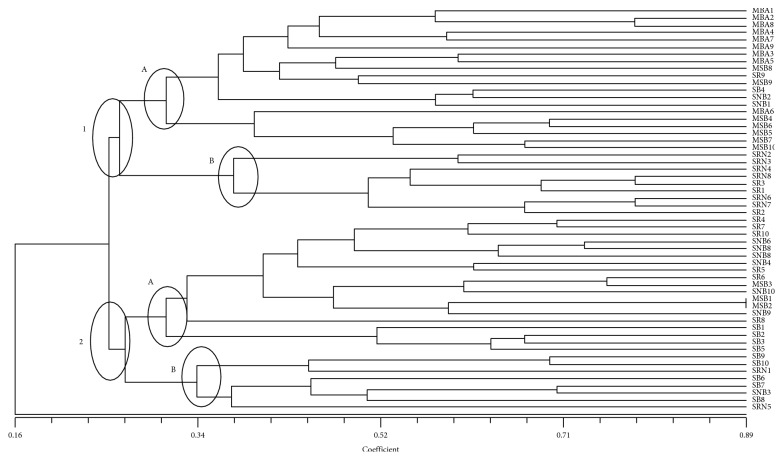
UPGMA dendrogram based on SSR profiles obtained from maize ecotypes. The Jaccard similarity index is indicated on the *X* axis. The sample names with their previously assigned (or not) genotype are reported on the *Y* axis (MBA = *Mais Bianco di Acerra*; SR = *Spiga Rossa*; SB = *Spiga Bianca*; SNB = *Spiga Napoletana Bianca*; SRN = *Spiga Rossa Napoletana*; MSB = *Mais Spogna Bianca*).

Accessions of six maize ecotypes were analysed to estimate genetic biodiversity and population structure. The SSR matrix was elaborated to perform a cluster analysis and obtain the dendrogram, as shown in Figure 1.

The dendrogram displays two macrogroups (1 and 2) connected with a node at JSC = 0.25. Each macrogroup is subdivided in two clusters (A and B) including samples of different ecotypes. In fact, in this analysis, samples of the same ecotype do not fall in well-separated groups. Each subgroup (1A, 1B, 2A, and 2B) includes not only many samples of the same ecotype but also individuals belonging to different ecotypes (*e.g.*, subgroup 1A includes the MBA samples and even some SNB and SB individuals).

The *H*_o_ and *H*_e_ values are similar for most of the analysed loci; moreover, in many cases, the Fixation Index (F) is close to zero, suggesting a random recombination among ecotypes and samples. The *F* value is greater than zero in the case of locus 6 or 5 relative to ecotypes MBA and SR. The high biodiversity is also confirmed by the PIC (min 0.3, max 0.4) and by the Shannon Index (I). In fact, the *I* values range from 0.00 to 1.28; the highest *I* value is observed for locus Phi029 in the case of MBA, the lowest for loci Nc130 and Phi014 in the case of SNR, and for locus Bnlg118 in the case of SR (Table 2).

**Figure 2:**
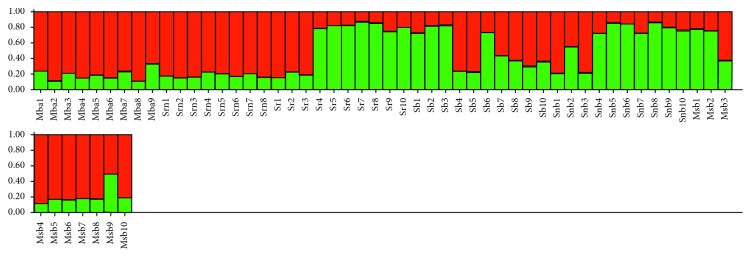
Bar plot of estimated membership probability (*Q*) for *K* = 2 for the SSR profiles of maize ecotypes. Sample names are indicated on the *X* axis. The estimated membership probability (*Q*) for *K* = 2 is represented on the *Y* axis.

Population structure was estimated by analysing the SSR dataset using structure software. The distribution of *∆Κ*, estimated by the Evanno method, identified a peak corresponding to *K* = 2. To investigate the substructure of the maize collection, the proportion of genotype membership *s* for *K* equal to 2 was computed (Figure 2).

At *K* = 2, the first cluster (red) includes the ecotypes *Mais Bianco di Acerra (*MBA), *Spiga Napoletana Rossa (*SNR), and *Spiga Bianca (*SB), whilst the samples belonging to *Spiga Rossa (*SR) and *Spiga Napoletana Bianca (*SNB) were grouped in the green cluster. However, the red cluster includes some admixed ecotypes, with a probability of belonging to the green cluster ranging from 20% to 80% as in the case of samples SB3 and SB9, respectively.

### 3.2. Onion

Four onion ecotypes, *Febbrarese (*F), *Marzatica (*M), *Ramata di Montoro (*R), and *Vatolla (*V), were analysed using seven microsatellite loci. The *H*_e_, *F*, and PIC values suggest that the studied onion ecotypes are not genetically stable. In fact, the positive values of *F* close to 1 indicate phenomena of intersection or null alleles, while negative values tending to −1 suggest an excess of heterozygosity (Table 3), probably due to the selection processes, or to an isolate-breaking effect (*i.e.*, the mixing of two previously isolated populations) in this region.

**Figure 3:**
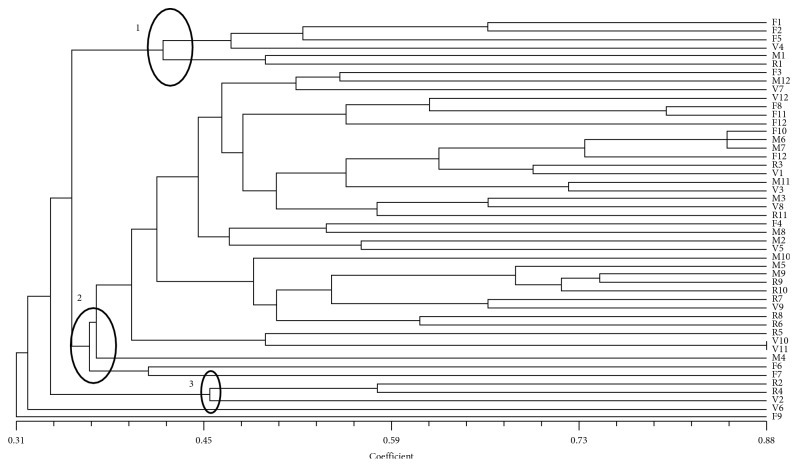
UPGMA dendrogram based on SSR profiles obtained from onion ecotypes. The Jaccard similarity index is indicated on the *X* axis. The sample names with their previously assigned (or not) genotype are reported on the *Y* axis (F = Febbrarese; M = Marzatica; R = Ramata di Montoro; V = Vatolla).

To estimate the genetic similarity among samples, an UPGMA analysis was performed on molecular data (Figure 3).

The JSC value is very low among different ecotypes, and thus the genetic biodiversity is very high; moreover, the ecotypes are distributed in admixed clusters. Clusters 1, 2, and 3 comprise many samples belonging to three different ecotypes (*Febbrarese*, *Marzatica*, and *Ramata di Montoro*). A similar result was obtained using a Bayesian statistical approach. The most probable *K* values are 6 and 8. At both *K* values, the obtained clusters appear admixed, and none of the samples can be clearly attributed to a specific cluster. In fact, they show the same probability of belonging to diverse clusters (data not shown).

### 3.3. Garlic

For the two investigated ecotypes of garlic, *Schiacciato* and *Tondo di Torella*, the *H*_o_ and *F* values suggest that, for all eight loci, there is an excess of heterozygosity. It is highly probable that the ecotypes were obtained by cross-breeding between each other (Table 4).

**Figure 4:**
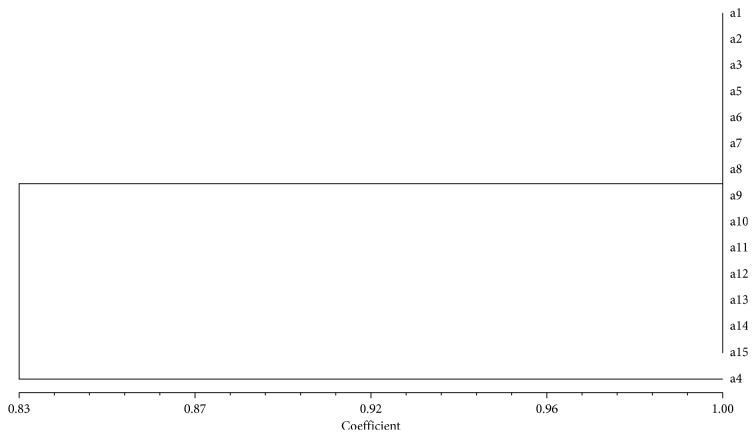
UPGMA dendrogram based on SSR profiles obtained from onion ecotypes. The Jaccard similarity index is indicated on the *X* axis. The sample names with their previously assigned (or not) genotype are reported on the *Y* axis (a1–a8 = Aglio Schiacciato; a9–a15 = Aglio Tondo di Torella).

The estimated JSC confirms that all analysed samples are identical, with the exception of a4 (0.87; Figure 4).

The molecular data are identical for all analysed samples, and therefore, the two ecotypes are not distinguishable using these SSR loci. The Bayesian bioinformatics elaboration was uninformative, and therefore, these results are not shown.

### 3.4. Escarole

The SSR analysis for the escarole ecotype revealed that A149, EU03H001, and B214 are homozygotic, and thus, the *F* index cannot be estimated for these loci because both *H*_o_ and *H*_e_ are equal to zero (Table 5).

The *F* values for loci A149 and EU003O are very high (close to 1) due to the fact that *H*_o_ is close to zero; *F* values indicate inbreeding events. *F* values for locus 4 are close to 0, as usually observed in the case of random reproduction; on the contrary, negative values for F, due to extreme heterozygosity and then to gene flow, are observed for loci B42 and EU01H08 (Table 5). Genetic biodiversity was estimated by calculating the probability of finding the same allele size in the population. This value is very low, the highest being that of loci B42 and EU01H08 (0.57 and 0.50, respectively). Moreover, the mean value of *I* is equal to 0.47, suggesting a limited genetic biodiversity (Table 5).

**Figure 5:**
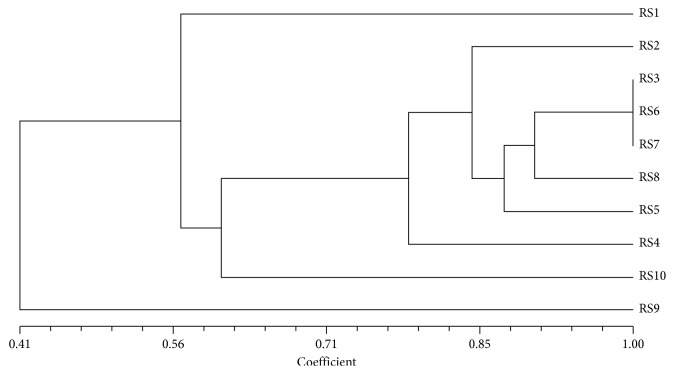
UPGMA dendrogram based on SSR profiles obtained from escarole ecotypes. The Jaccard similarity index is indicated on the *X* axis. The sample names with their previously assigned (or not) genotype are reported on the *Y* axis (RS = Riccia Schiana).

The cluster analysis dendrogram (Figure 5) reveals that the escarole ecotypes are characterised by a high genetic biodiversity; in particular, all samples, with the exception of 1, 9, and 10, show a genetic similarity >85%.

### 3.5. Courgette

The biodiversity indices for the two ecotypes of courgette are shown in Table 6. The mean values of *I* are 0.54 and 0.57 for *Cilentano (*C) and *San Pasquale (*S), respectively. However, this is not valid for some loci. For instance, in the case of *Cilentano*, loci 4991 and 5800 show higher values as do loci 1906, 4991, and 5800 for *San Pasquale* (Table 6). In agreement with these results, the *F* values varied from negative (*San Pasquale*, loci 4991 and 5800) to positive (*Cilentano*, loci 1906 and 4782).

**Figure 6:**
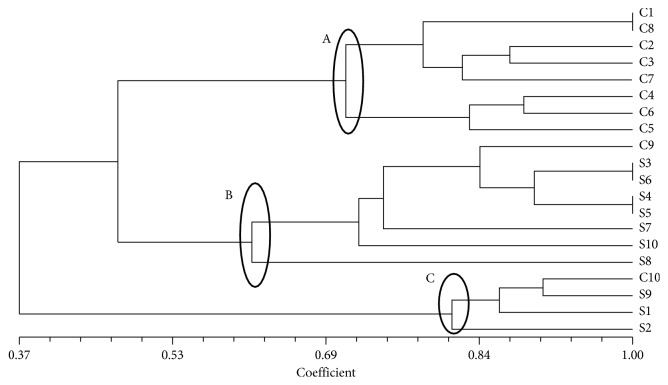
UPGMA dendrogram based on SSR profiles obtained from couguette ecotypes. The Jaccard similarity index is indicated on the *X* axis. The sample names with their previously assigned (or not) genotype are reported on the *Y* axis (C = Cilentano; S = San Pasquale).

The UPGMA dendrogram reveals three clusters (Figure 6): cluster A includes samples z1 to z8; cluster B comprises sample z9, samples z10 to z18, and sample z20; cluster C includes samples z10 to z12 and sample z19 and was dissimilar from others analysed samples (0.37).

**Figure 7:**
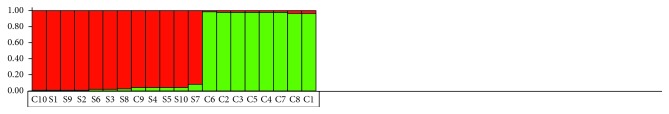
Bar plot of estimated membership probability (*Q*) for *K* = 2 for the SSR profiles of courguette ecotypes. Sample name are indicated on the *X* axis. The estimated membership probability (*Q*) for *K* = 2 is represented on the *Y* axis (C = Cilentano; S = San Pasquale).

The population structure analysis performed on these molecular data shows that the optimal population numbers (K) are 2 and 3 (Figure 7).

For *K* = 2, samples are well separated into two clusters (green and red). However, samples C9 and C10, belonging to *Cilentano*, fall within the cluster including all accessions of *San Pasquale*. For *K* = 3 (data not shown), the resulting clusters are well separated. Samples C1 to C8 of *Cilentano* are allocated to the red cluster, while all other samples are distributed in two groups: green and blue. The *San Pasquale* samples C10, S1, S1, and S9 are separate from the others (blue cluster). Moreover, some samples belonging to *San Pasquale* (green cluster) show a 20% probability of being assigned to the blue cluster (*e.g.*, S4 and S6).

## 4. Discussion

The Mediterranean Basin is one of the rarest terrestrial biomes, covering a mere 2% of Earth's land surface [21]. Its small size is compensated by a high biodiversity since over 20% of known vascular plant taxa are present in this biome [22], and many of which are exceedingly rare and considered as endemisms [23]. Moreover, the Mediterranean region, due to its mild climate, is inhabited by millions of people.

Because of its peculiar pedoclimatic conditions, the Mediterranean region has also been the cradle of the most important horticultural crops. In fact, many endemic species persist only on small remnants of their natural habitat separated by intensively cultivated agricultural land, thereby contributing to ecotype development. These ecotypes have an economical, ecological, and biological role. In this work, we estimated the genetic biodiversity of several ecotypes belonging to five different horticultural species: maize, onion, garlic, escarole, and courgette, occurring in the Campania Region (southern Italy). The final objective is to preserve the horticultural Campania ecotypes by means of in situ and/or ex situ conservation strategies. When the project started, the investigated ecotypes were not genetically characterized, although some of that, and in particular onion, was of interest from organoleptic and economic point of view. Therefore, no program of conservation in situ or ex situ was in progress. For this reason, the data reported in this manuscript are very useful to begin a program of conservation in situ, through the keeper farmers, and, ex situ, maintaining the genetic resources in the Universities and/or Research Centres.

### 4.1. Maize

Maize has a strategic importance for agricultural and food of all industrialized countries. In addition to human consumption, its grain is also used for raising cattle [24]. In this context, the biodiversity study and germplasm characterization of local ecotypes is needed to adopt conservation and protection strategies as well as the exploitation of local genetic resources, which are essential to improve the quality, durability, and sustainability of horticulture [25]. This randomly selects half the genes from a given plant to propagate to the next generation, meaning that desirable traits found in the crop (like high yield or good nutrition quality) might be lost in subsequent generations unless certain breeding techniques are used to keep and maintain these superior characters. Maize reproduces sexually each year and had endosperms that ranged in the ploidy level from diploid (2x) to nonaploid (9x). In crosses with diploid males, only kernels of the triploid endosperm class developed normally.

In this study, we investigated six ecotypes of maize through seven SSR markers. The *H*_o_ and *H*_e_ mean values of the analysed loci were similar; moreover, in many cases, the *F* value is close to zero, suggesting a random recombination among different ecotypes. An average of six alleles for each locus were found, in agreement with the data reported for the same analysed loci by Ranatunga et al. [26] and Pejic et al. [27]; on the contrary, Molin et al. [28] observed an average of about three alleles per locus. The PIC varied in relation to the locus; however, these values were similar to those reported by Adeyemo et al. [29]. In that case, the authors observed a high genetic diversity among maize plants, as highlighted by the genetic distance, and the intraecotype molecular variance was about twice the interecotype one. Our results are in agreement with those obtained by Adeyemo et al. [29] although some differences were detected. Smith et al. [30] analysed loci phi014, phi024, and phi029 and found PIC values equal to 0.20, 0.55, and 0.67, respectively. In the case of phi014, our data are similar to those reported by Smith et al. [30] for ecotypes *Spiga Rossa* and *Spogna Bianca*, while in the case of ecotypes *Bianco di Acerra*, *Spiga Bianca*, and *Spiga Napoletana Bianca,* the PIC we obtained is higher. In the case of loci phi024 and phi029, the PIC values are lower than those reported by the same authors for inbred lines, suggesting a lower genetic variability in these loci relative to the inbred lines analysed in other studies. Although the genetic biodiversity of maize has been the object of several studies, this is the first one addressing the Campania Region ecotypes. A biodiversity study was conducted by Legesse et al. [14] to improve the knowledge on genetic diversity and relationships among maize inbred lines to sustain a breeding program. In that case, 56 high land and midaltitude maize inbred lines were genotyped using SSR loci and these genotypes were clearly distinguishable. On the contrary, maize ecotypes analysed by us were admixed and not clearly distinguishable. Despite the high molecular variance existing among the ecotypes, the population structure analysis grouped the samples into two broad clusters. Structure results showed that the best clustering of all samples was when they were split into two groups because of their high genetic biodiversity, which does not allow a clear separation of populations for *K* values higher than two. This result was confirmed by the graphical representation of the genetic similarity. In the similarity dendrogram, in fact, the samples were separated in two main groups, suggesting that the six maize ecotypes were not stabilized lines. The intraecotype genetic biodiversity was greater than the interecotype one. Some ecotypes, which might seem synonymies because of their names, were, on the contrary, clearly separated. In particular, samples belonging to ecotype *Mais Bianco di Acerra* were grouped in different clusters from those formed by the ecotypes *Spiga Napoletana Bianca* and *Spiga Bianca*, which in turn were divided into two subgroups. However, a basal cluster can be observed, in which samples belonging to different ecotypes were allocated. Our results confirm that the maize ecotypes are not stabilized lines and represent an important source of genetic biodiversity useful for further breeding programmes.

### 4.2. Onion

Onions are the second most produced horticulture crop after tomatoes, with about 3'642'000 ha grown annually worldwide and a production of 53.6 Mt. (2017). Their large distribution is probably due to the versatile culinary uses as the raw food or in different manners of cooking (baked, boiled, braised, grilled, fried, and roasted). In addition, onions are among the healthier vegetables due to the high levels of bioactive compounds, such as phenolic acids, flavonoids, thiosulfinates, and anthocyanins.

The flowers are proterandrous, the male flower matures before the female one; moreover, fertilization is allogamous, favoured by pollinating insects.

The *H*_e_, Fixation Index, and PIC values highlighted that the onion ecotypes analysed were not genetically stabilized. In fact, the *F* values suggest phenomena of out-crossings, null alleles, or an excess of heterozygosity probably due to the selection techniques designed to enhance the heterosis phenomenon [31]. Selection techniques are commonly exploited by genetic hybridisation programs and consist in an event where two distinct genomes undergo a phase of “genetic turbulence” before entering a phase of homeostasis. This stage of “Genomic Shock,” proposed by McClintock [32], may be in relation to gene expression and with the activation of transposons [32]. Onion reproduces exclusively by sexual fertilization, favoured by insect pollinators. This feature could explain the fact that *H*_o_ values are greater than those of *H*_e_. However, present results are in line with those reported by Baldwin et al. [33]. For most of the investigated loci, the gene flow was compatible with natural and random gene flow phenomena (loci 1, 3, and 5 of *Febbrarese*). The D_est_ values also support this hypothesis; in fact, they range from a minimum of 0 at locus 2, to a maximum 0.55 at locus 4. Onion often undergoes self-pollination phenomena; in fact, the ecotypes showed a significant inbreeding depression, even higher than that reported by Vigouroux et al. [34] for corn, which is considered one of the most tolerant species with respect to this phenomenon. The level of pollination due to out-crossing phenomena was assayed by Van Der Meer and van Bennekom [35] using two onion varieties, a yellow bulb (recessive trait), against a red bulb (dominant character), and the results showed that, over the four years of study, it could vary from 29 to 92% in relation to climatic and environmental changes that occur seasonally. The bioinformatics analyses performed on the onion ecotypes of Campania confirm not only the remarkable genetic variability but also a probable high genetic rearrangement [36].

### 4.3. Garlic

In the case of garlic, all the biodiversity indices and the population structure analyses of the two ecotypes, *Schiacciato* and *Tondo di Torella*, suggest that they are genetically identical. Therefore, a case of synonymy is possible; moreover, garlic flowers as well as seeds are sterile. Its propagation and production is obtained by means of vegetative reproduction by division and planting of cloves [37]. Garlic genotypes can adapt to different agroclimatic regions [38] and although it is cultivated and propagated worldwide, it probably originated in Central Asia [39]. Our study is the first to investigate the Italian and, in particular, Campania ecotypes of garlic and shows that these do not exhibit molecular variance. Our data, unlike those obtained by Zhao et al. [16], who analysed the same SSR loci in different garlic varieties, show no hypervariability; the mean PIC value is about half of that obtained by Zhao et al. [16], confirming the small genetic biodiversity existing between these two ecotypes of garlic. The similarity analysis also revealed that the JSC was equal to one, with the exception of sample a4, which showed a similarity of 0.83. This lower similarity might be related to a single allele present at SSR locus 40, probably caused by somatic mutation, a common phenomenon for vegetatively propagated crops.

### 4.4. Escarole

Escarole is diploid (2n = 18) and allogamous [40]; its floral morphology favours genetic exchange. This species has long been used as a medicinal plant [41], and it is now cultivated for several different purposes, including food. Molecular analyses, performed on 10 plants of the *Riccia Schiana* ecotype, revealed that there is a discrete genetic variability among the investigated specimens, probably favoured by its reproductive mechanism. The *H*_o_ mean value was very low, reflecting the high homozygosity of the investigated loci; the Shannon Index suggested that the intraecotype biodiversity was limited. The similarity dendrogram showed that the analysed specimens constituted a fairly homogeneous group with high similarity values (about 85%), with the exception of samples RS1, RS9, and RS10. Genetic variability is affected by a number of factors including out-crossing mechanisms, environment, and natural or artificial selection [42]. In fact, Azevedo et al. [43] reported that the genetic variation distribution of escarole is not random within populations and is determined by the reproductive system, geographic distribution, effective scale of populations, pattern of reproduction, gene flow through the spread of pollen, and evolutionary factors [44]. Italy is probably one of the sites of origin of the genus *Cichorium* [45]. Therefore, it is necessary to protect the biodiversity of this germplasm and introduce new genotypes from regions with a high genetic biodiversity for further breeding programmes. However, a broader genetic basis and potential gene resources with resistances to biotic and abiotic stresses can be obtained through breeding of wild varieties [46].

### 4.5. Courgette

Courgette is cultivated all over the world as different varieties and ecotypes well adapted to diverse climates and environments [47]. Zucchini requires plentiful bees for pollination. In areas where pollinator decline or high amount of pesticides is used, such as mosquito-spray districts, gardeners often experience fruit abortion, where the fruit begins to grow and then dries or rots. This is due to an insufficient number of pollen grains delivered to the pistil. It can be corrected by hand pollination or by increasing the bee population. The organoleptic characteristics of courgette are related to the environment in which they are grown and to agricultural practices. Ecotypes *Cilentano* and *San Pasquale* have low *H*_o_ values for all analysed loci, in line with the H_e_, although *San Pasquale* showed the highest heterozygosity values in the case of loci 1 and 7. The *I* and PIC values confirmed that the two ecotypes are not characterized by a high genetic biodiversity, probably because of an ancestral selection aimed at the genetic and phenotypic stabilization of the lines [48]. In fact, the PIC mean values were approximately half of those observed by Barzegar et al. [49], while *I* was about one-third. The dendrogram revealed that courgette ecotypes had a high genetic similarity, especially in the case of *Cilentano*. Barzegar et al. [49], estimating the genetic biodiversity of 26 accessions of *C. pepo* by means of microsatellites, reported similarity values with a maximum equal to 0.5 on a scale from 0 to 1. The number of alleles per locus obtained in our study varies from a minimum of one to a maximum of five, in agreement with the data obtained by Paris et al. [50]. Yu et al. [51] reported that the number of alleles, detectable through SSR markers, is directly related to the number of repetitions because microsatellite sequences are usually hypervariable, and it is reasonable to expect that they should occur more frequently in noncoding regions than in coding ones, especially for those with di- or tetranucleotide motifs. In our study, the length of the repetitions varies from six to eight nucleotide bases, but locus 2 was detected despite being monomorphic. The remarks made by Yu et al. [51] about the SSR marker and above reported are contradicted to some extent by Barzegar et al. [49] who showed that a greater number of polymorphic alleles are dinucleotide repeat (AG)8 in *C. pepo*.

Breeding of compatible, multidisease-resistant ecotypes with tolerance to abiotic stresses is crucial for the sustainable production of courgette. Knowledge on the genetic biodiversity present in ecotypes is of fundamental importance, not only for the genetic improvement but also for cultivar identification and protection. SSR markers have been extensively used for commercial cultivar discrimination and assessment of genetic biodiversity of commercial cultivars [52]. Kong et al. [53] reported that SSR markers were used to determine the genetic diversity and relationships of 35 *Cucurbita* rootstock accessions. In conclusion, the molecular analyses conducted on courgette ecotypes of Campania highlight a good stabilization aimed at obtaining homogeneous lines [54].

## 5. Conclusions

The results reported here reveal, for the first time, that a high genetic biodiversity is present in Italian horticultural crops, in particular those grown in the Campania Region. The ancient traditions of this region have allowed maintaining several agricultural ecotypes with a high genetic biodiversity. This feature is very important in view of climate change and for sustaining improved quality and yield in order to produce agricultural products of excellence with a very important social and economic role. At the present, also on the basis of these results, the Campania Region has planned a series of initiatives to preserve the agrobiodiversity, and some of them are carrying out. The main aim of the study is to ensure that biodiversity can contribute to the development and enhancement of rural areas, especially those where a high crop biodiversity is present and has to be maintained. In this sense, the regional programs foresee the recovery and enhancement of the ecotypes of autochthonous species, as well as safeguard the environment, in the broader perspective of protecting the typical local agriproducts. Starting from these and other results, the Campania region has adopted a model based mainly on the Regional repertoire of the main endangered genetic resources, by means of keeper farmers, germplasm banks, conservation, and safety network.

## Figures and Tables

**Table 1:** List of the analysed ecotypes.

Species	Ecotype	Abbreviation
*Allium sativum* L.	“Tondo di Torella”	a9-a15
*Allium sativum* L.	“Schiacciato”	a1-a8
*Allium cepa* L.	“Febbrarese”	F
*Allium cepa* L.	“Cipolla Marzatica”	M
*Allium cepa* L.	“Ramata di Montoro”	R
*Allium cepa* L.	“Vatolla”	V
*Zea mays* L.	“Bianco di Acerra”	MBA
*Zea mays* L.	“Spiga Bianca”	SB
*Zea mays* L.	“Spiga Napoletana Bianca”	SNB
*Zea mays* L.	“Spiga Napoletana Rossa”	SNR
*Zea mays* L.	“Spiga Rossa”	SR
*Cichorium endivia* L.	“Riccia Schiana”	RS
*Cucurbita pepo* L.	“Cilentano”	C
*Cucurbita pepo* L.	“San Pasquale”	S

**Table 2:** Genetic indices calculated for the SSR pattern.

Ecotype	Locus	*N*	*N*_a_	*N*_e_	*I*	*H*_o_	*H*_e_	UH_e_	*F*	Gene diversity	PIC
“Bianco di Acerra”	Bnlg118	10	4	2.86	1.16	0.6	0.65	0.68	0.08	0.67	0.6
	Bnlg391	10	3	2.25	0.94	0.3	0.56	0.58	0.46	0.56	0.49
	Nc130	9	2	1.12	0.21	0.11	0.1	0.11	−0.06	0.1	0.1
	Nc133	10	2	1.47	0.5	0.2	0.32	0.34	0.38	0.32	0.27
	Phi014	10	4	2.15	1.03	0.5	0.54	0.56	0.07	0.54	0.5
	Phi024	10	4	2.11	0.98	0.2	0.53	0.55	0.62	0.53	0.48
	Phi029	5	4	3.33	1.28	0.8	0.7	0.78	−0.14	0.7	0.65

Mean value		9	3	2.18	0.87	0.39	0.49	0.51	0.2	0.49	0.44

“Spiga Napoletana Rossa”	Bnlg118	5	3	2.27	0.95	0.4	0.56	0.62	0.29	0.56	0.5
	Bnlg391	10	2	2	0.69	0	0.5	0.53	1	0.5	0.38
	Nc130	8	1	1	0	0	0	0	#N/D	0	0
	Nc133	10	4	2.6	1.09	0.6	0.62	0.65	0.02	0.62	0.54
	Phi014	10	1	1	0	0	0	0	#N/D	0	0
	Phi024	8	4	2.72	1.14	1	0.63	0.68	−0.58	0.63	0.57
	Phi029	7	2	1.69	0.6	0.57	0.41	0.44	−0.4	0.41	0.32

Mean value		8	2	1.9	0.64	0.37	0.39	0.42	#N/D	0.39	0.33

“Spiga Rossa”	Bnlg118	8	1	1	0	0	0	0	#N/D	0	0
	Bnlg391	10	3	2.06	0.82	0.6	0.52	0.54	−0.17	0.52	0.42
	Nc130	8	2	2	0.69	0	0.5	0.53	1	0.5	0.38
	Nc133	10	3	2.99	1.1	0.7	0.67	0.7	−0.05	0.67	0.59
	Phi014	8	2	1.28	0.38	0	0.22	0.23	1	0.22	0.19
	Phi024	9	3	2.22	0.87	0.56	0.55	0.58	−0.01	0.55	0.45
	Phi029	8	2	1.44	0.48	0.38	0.3	0.33	−0.23	0.3	0.26

Mean value		8	2	1.86	0.62	0.32	0.39	0.42	#N/D	0.39	0.33

“Spiga Bianca”	Bnlg118	9	4	2.57	1.12	0.67	0.61	0.65	−0.09	0.61	0.56
	Bnlg391	10	4	2.74	1.14	0.4	0.64	0.67	0.37	0.64	0.57
	Nc130	7	3	2.33	0.96	0.57	0.57	0.62	0	0.57	0.5
	Nc133	10	3	2.9	1.08	0.6	0.66	0.69	0.08	0.66	0.58
	Phi014	8	3	2.67	1.04	0.63	0.63	0.67	0	0.63	0.55
	Phi024	10	3	1.8	0.75	0.6	0.45	0.47	−0.35	0.45	0.38
	Phi029	10	2	1.1	0.2	0.1	0.1	0.1	−0.05	0.1	0.09

Mean value		9	3	2.3	0.9	0.51	0.52	0.55	−0.01	0.52	0.46

“Spiga Napoletana Bianca”	Bnlg118	9	4	3.06	1.21	0.89	0.67	0.71	−0.32	0.67	0.61
	Bnlg391	9	4	2.79	1.16	0.89	0.64	0.68	−0.38	0.64	0.58
	Nc130	9	2	1.53	0.53	0.44	0.35	0.37	−0.29	0.35	0.29
	Nc133	9	2	1.25	0.35	0.22	0.2	0.21	−0.13	0.2	0.18
	Phi014	9	3	2.05	0.83	0.11	0.51	0.54	0.78	0.51	0.43
	Phi024	9	3	2.16	0.9	0.56	0.54	0.57	−0.03	0.54	0.47
	Phi029	8	3	2.84	1.07	0.5	0.65	0.69	0.23	0.67	0.61

Mean value		9	3	2.24	0.86	0.52	0.51	0.54	−0.02	0.51	0.45

“Spogna Bianca”	Bnlg118	5	4	2.94	1.22	0.8	0.66	0.73	−0.21	0.66	0.61
	Bnlg391	8	4	3.12	1.25	0.88	0.68	0.73	−0.29	0.68	0.62
	Nc130	8	2	1.13	0.23	0.13	0.12	0.13	−0.07	0.12	0.11
	Nc133	8	2	1.97	0.69	0.63	0.49	0.53	−0.27	0.49	0.37
	Phi014	7	2	1.15	0.26	0.14	0.13	0.14	−0.08	0.26	0.24
	Phi024	7	2	1.15	0.26	0.14	0.13	0.14	−0.08	0.13	0.12
	Phi029	8	3	2.72	1.04	0.88	0.63	0.68	−0.38	0.63	0.56

Mean value		7	3	2.03	0.71	0.51	0.41	0.44	−0.2	0.42	0.38

*N* = number of alleles; *N*_e_ = number of effective alleles; *I* = Shannon Index; *H*_o_ = observed heterozygosity; *H*_e_ = expected heterozygosity; UH_e_ = unbiased expected heterozygosity; *F* = Fixation Index; PIC = polymorphic index content.

**Table 3:** Genetic indices calculated for the SSR pattern.

Ecotype	Locus	*N*	*N*_a_	*N*_e_	*I*	*H*_o_	*H*_e_	UH_e_	*F*	Gene diversity	PIC
Febbrarese	AMS03	8	6	3.76	1.54	0.75	0.73	0.78	−0.02	0.73	0.7
	AMS08	9	1	1	0	0	0	0	NA	0	0
	AMS13	7	2	1.32	0.41	0.29	0.24	0.26	−0.17	0.24	0.21
	ACM132	7	7	5.44	1.81	0.57	0.82	0.88	0.3	0.82	0.79
	ACAEM68	8	2	1.88	0.66	0.5	0.47	0.5	−0.07	0.47	0.36
	ACAFC04	8	3	2.51	0.98	0.88	0.6	0.64	−0.45	0.6	0.52
	ACACL08	8	4	3.46	1.31	0.88	0.71	0.76	−0.23	0.71	0.66

Mean value		8	4	2.77	0.96	0.55	0.51	0.55	NA	0.51	0.46

Marzatica	AMS03	4	5	3.2	1.39	0.75	0.69	0.79	−0.09	0.69	0.65
	AMS08	6	1	1	0	0	0	0	NA	0	0
	AMS13	6	2	1.8	0.64	0.67	0.44	0.48	−0.5	0.44	0.35
	ACM132	6	4	3	1.24	0.33	0.67	0.73	0.5	0.67	0.62
	ACAEM68	5	2	1.47	0.5	0	0.32	0.36	1	0.32	0.27
	ACAFC04	5	2	2	0.69	1	0.5	0.56	−1	0.5	0.38
	ACACL08	5	2	1.92	0.67	0.8	0.48	0.53	−0.67	0.48	0.36

Mean value		5	3	2.06	0.73	0.51	0.44	0.49	NA	0.44	0.38

Ramata di Montoro	AMS03	11	6	3.51	1.45	0.82	0.71	0.75	−0.14	0.75	0.71
	AMS08	11	1	1	0	0	0	0	NA	0.1	0.09
	AMS13	10	3	1.5	0.61	0.4	0.34	0.35	−0.19	0.44	0.41
	ACM132	9	6	4.05	1.57	0.67	0.75	0.8	0.11	0.78	0.75
	ACAEM68	11	7	2.95	1.46	0.45	0.66	0.69	0.31	0.75	0.72
	ACAFC04	11	3	2.07	0.82	0.82	0.52	0.54	−0.58	0.57	0.49
	ACACL08	10	5	3.39	1.35	0.6	0.71	0.74	0.15	0.72	0.67

Mean value		10	4	2.64	1.04	0.54	0.53	0.55	NA	0.59	0.55

Vatolla	AMS03	9	3	2.22	0.87	0.89	0.55	0.58	−0.62	0.55	0.45
	AMS08	10	1	1	0	0	0	0	NA	0	0
	AMS13	9	2	1.91	0.67	0.78	0.48	0.5	−0.64	0.44	0.35
	ACM132	9	5	3.68	1.43	0.67	0.73	0.77	0.08	0.77	0.73
	ACAEM68	9	5	3.24	1.34	0.56	0.69	0.73	0.2	0.66	0.61
	ACAFC04	10	2	1.92	0.67	0.8	0.48	0.51	−0.67	0.48	0.36
	ACACL08	10	3	2.74	1.05	0.5	0.64	0.67	0.21	0.65	0.58

Mean value		9	3	2.39	0.86	0.6	0.51	0.54	NA	0.51	0.44

*N* = Number of alleles; *N*_e_ = number of effective alleles; *I* = Shannon Index; *H*_o_ = observed heterozygosity; *H*_e_ = expected heterozygosity; UH_e_ = unbiased expected heterozygosity; *F* = Fixation Index; PIC = polymorphic index content.

**Table 4:** Genetic indices calculated for the SSR pattern.

Ecotype	Locus	*N*	*N*_a_	*N*_e_	*I*	*H*_o_	*H*_e_	UH_e_	*F*	Gene diversity	PIC
Schiacciato	35	10	2	1.98	0.69	0.9	0.5	0.52	−0.82	0.5	0.375
	40	10	3	2.2	0.86	1	0.55	0.57	−0.83	0.5	0.375
	53	10	2	2	0.69	1	0.5	0.53	−1	0.5	0.375
	59	10	2	2	0.69	1	0.5	0.53	−1	0.5	0.375
	72	10	2	2	0.69	1	0.5	0.53	−1	0.5	0.375
	80	10	2	2	0.69	1	0.5	0.53	−1	0.5	0.375

Mean value		10	2.17	2.03	0.72	0.98	0.51	0.54	−0.94	0.50	0.38

Tondo di Torella	35	5	2	1.92	0.67	0.8	0.48	0.53	−0.67	0.5	0.375
	40	5	2	2	0.69	1	0.5	0.56	−1	0.5	0.375
	53	5	2	2	0.69	1	0.5	0.56	−1	0.5	0.375
	59	5	2	2	0.69	1	0.5	0.56	−1	0.5	0.375
	72	5	2	2	0.69	1	0.5	0.56	−1	0.5	0.375
	80	5	2	2	0.69	1	0.5	0.56	−1	0.5	0.375

Mean value		5	2	1.99	0.69	0.97	0.50	0.56	−0.95	0.5	0.375

*N* = number of alleles; *N*_e_ = number of effective alleles; *I* = Shannon Index; *H*_o_ = observed heterozygosity; *H*_e_ = expected heterozygosity; UH_e_ = unbiased expected heterozygosity; *F* = Fixation Index; PIC = polymorphic index content.

**Table 5:** Genetic indices calculated for the SSR pattern.

Ecotype	Locus	*N*	*N*_a_	*N*_e_	*I*	*H*_o_	*H*_e_	UH_e_	*F*	Gene diversity	PIC
Riccia Schiana	A149	10	2	1.47	0.5	0	0.32	0.34	1	0.32	0.27
	EU03H01	8	1	1	0	0	0	0	NA	0	0
	EU0030	9	3	1.74	0.73	0.11	0.43	0.45	0.74	0.43	0.37
	EU03D01	10	2	1.11	0.2	0.1	0.1	0.1	−0.05	0.1	0.09
	Sw2h09.2	9	3	1.59	0.68	0.22	0.37	0.39	0.4	0.37	0.34
	B214	10	1	1	0	0	0	0	NA	0	0
	B42	10	4	2.33	1	0.8	0.57	0.6	−0.4	0.57	0.49
	EU01H08	10	2	1.98	0.69	0.9	0.5	0.52	−0.82	0.5	0.37

Mean value		9.5	2.25	1.53.	0.47	0.27	0.28	0.3	NA	0.28	0.24

*N* = number of alleles; *N*_e_ = number of effective alleles; *I* = Shannon Index; *H*_o_ = observed heterozygosity; *H*_e_ = expected heterozygosity; UH_e_ = unbiased expected heterozygosity; *F* = Fixation Index; PIC = polymorphic index content.

**Table 6:** Genetic indices calculated for the SSR pattern.

Ecotype	Locus	*N*	*N*_a_	*N*_e_	*I*	*H*_o_	*H*_e_	UH_e_	*F*	Gene diversity	PIC
Cilentano	1906	9	3	1.26	0.43	0.11	0.2	0.22	0.45	0.2	0.19
	4307	10	2	1.22	0.33	0.2	0.18	0.19	−0.11	0.18	0.16
	4399	10	1	1	0	0	0	0	NA	0	0
	4782	10	2	1.22	0.33	0	0.18	0.19	1	0.18	0.16
	4991	10	4	2.9	1.17	0.9	0.66	0.69	−0.37	0.66	0.59
	5739	6	1	1	0	0	0	0	NA	0	0
	5800	8	5	4.41	1.54	0.75	0.77	0.83	0.03	0.77	0.74

Mean value		9	3	1.86	0.54	0.28	0.28	0.3	NA	0.28	0.26

San Pasquale	1906	10	4	3.45	1.3	1	0.71	0.75	−0.41	0.71	0.66
	4307	10	1	1	0	0	0	0	NA	0	0
	4399	10	1	1	0	0	0	0	NA	0	0
	4782	10	2	1.47	0.5	0.2	0.32	0.34	0.38	0.32	0.27
	4991	10	4	2.82	1.16	0.9	0.65	0.68	−0.4	0.65	0.59
	5739	9	1	1	0	0	0	0	NA	0	0
	5800	10	4	2.44	1.02	1	0.59	0.62	−0.69	0.59	0.5

Mean value		10	2	1.88	0.57	0.44	0.32	0.34	NA	0.32	0.29

*N* = number of alleles; *N*_e_ = number of effective alleles; *I* = Shannon Index; *H*_o_ = observed heterozygosity; *H*_e_ = expected heterozygosity; UH_e_ = unbiased expected heterozygosity; *F* = Fixation Index; PIC = polymorphic index content.

## Data Availability

The binary matrices data used to support the findings of this study have been deposited in the GitHub repository, and they will be made public after publication at https://github.com/fragua1804/agrigenet.

## Supplementary Materials

Supplementary Materials Table SM1: primers used for the SSR amplifications.
