# Supplementary material for: Ecotype-Level Genetic Biodiversity of Five Italian Traditional Crops
Source: Scientifica (Cairo). 2019 Jul 1;2019:4652769. doi: 10.1155/2019/4652769 (PMC6636500; doi:10.1155/2019/4652769)
Supplement: Supplementary Materials — Table SM1: primers used for the SSR amplifications. [file 4652769.f1.docx]

Table SM1

Table of the primers used for the SSR amplifications.

| **Plant species** | **Primer FW** | **Primer REV** | **Annealing**  **Tempetature (°C)** |
| --- | --- | --- | --- |
| ***Garlic*** |  |  |  |
| 35 | TTGGACTGAATTCTGAATACCT | GGGTGTGTGGTTCAAGGA | 55 |
| 40 | CACAGCAACATGCACCAT | TGCCGGAACTCGATATT | 54 |
| 53 | ACAAGGTCGACATCGTTTG | GGGCTTCACCTGAACACA | 55 |
| 59 | CTTGCCGGAACTCGATATT | CACAGCAACATGCACCAT | 55 |
| 72 | CACGCGAATCTTTCTTGG | TGCAAAGCAATATGGCAG | 54 |
| 80 | AATCTCCCTCCAAAGTCC | CCTGTATTTTGTGTAAAGCATCA | 57 |
|  |  |  |  |
| ***Onion*** |  |  |  |
| AMS03 | TAACCCTAGGATGAGTTGAG | GGATTTCCTCTTGAGATGA | 55 |
| AMS08 | GCCACGATGTTGAGATTTCG | CCCGAATATCCCACCAGTTC | 52 |
| AMS13 | ACCTTTTAAATTGACGATATTCC | CTGCACTATTCTGTGATGTATTTC | 57 |
| ACM132 | ATGGGGCCTGGTAAGTTTTT | TGCACACCGTTTCCATTTTA | 59 |
| ACAEM68 | ACGGTTTGATGCACAAGATG | CCAACCAACAGTTGATACTGC | 54 |
| ACAFC04 | TTTCAGCAACATAGTATTGCGTC | TCTTCGGGATTGGTATGGAG | 58 |
| ACACL08 | CCACATGGATGAAAAACACAA | CGCTGGTAGCTGAAGCAAAT | 60 |
|  |  |  |  |
| ***Mays*** |  |  |  |
| Bnlg118 | CTTCCAGCCGCAACCCTC | CCAACAACGCGGACGTGA | 61 |
| Bnlg391 | CAGATATCACAGCATCAGAAGATCA | AAAATGTAAGAACTTGTTTGGGATT | 60 |
| Nc130 | GCACATGAAGATCCTGCTGA | TGTGGATGACGGTGATGC | 57 |
| Nc133 | AATCAAACACACACCTTGCG | GCAAGGGAATAAGGTGACGA | 57 |
| Phi014 | AGATGACCAGGGCCGTCAACGAC | CCAGCTTCACCAGCTTGCTCTTCGTG | 68 |
| Phi024 | ACTGTTCCACCAAACCAAGCCGAGA | AGTAGGGGTTGGGGATCTCCTCC | 66 |
| Phi029 | TTGTCTTTCTTCCTCCACAAGCAGCGAA | ATTTCCAGTTGCCACCGACGAAGAACTT | 65 |
|  |  |  |  |
| ***Escarole*** |  |  |  |
| A149 | CTGCTATGGACAGTTCCAGT | CAATTCAGTTGTGATAGACGC | 57 |
| EU03H01 | GCCATTCCTTTCAAGAGCAG | AACCCAAAACCGCAACAATA | 61 |
| EU0030 | AGCACGACTCTGCTGCTCTTTTT | CGAGCCATGTTAGGGTTTGT | 59 |
| EU03D01 | ATGTCGGAGCAAAATCGTTC | CATGTTCCCGCTCATGAATA | 57 |
| Sw2h09.2 | GTGCCGGTCTTCAGGTTACA | CGCCTACCGATTACGATTGA | 54 |
| B214 | AAAGTCACACATCGCATTTCCT | GTAGCAGCAGCAGCCATCTT | 55 |
| B42 | GGAGCAGGTAGAGTCCCATC | CGTTTGAAAATTTATACCAAAATG | 58 |
| EU01H08 | TTCGAGTCTTGCCTTAATTGTT | CAGACGACCTTACGGCAACT | 59 |
|  |  |  |  |
| ***Courgette*** |  |  |  |
| 1906 | GGAGGAGGGATAGAGAGAGAAACA | ACGATTTCTTCAGCCCTCAAAG | 63 |
| 4307 | GAACCTCGTTGCTGGTTCTTCT | TCTAGCATCTTACGCACGCTTC | 57 |
| 4399 | TGTCAAATTCGCTTCCATCATC | ACGACTGTGAGAACGGTGAAAA | 57 |
| 4782 | TGGAATCCAGAGACTGATGAAG | CACGATTCCGATAACAACAAGA | 60 |
| 4991 | TGGAGGTATCCTTCCGTATGTT | CATACGGGAGTTTCGTTTTTCT | 60 |
| 5739 | CTTCTCTACCGGACCCTCCTAC | CTCAAGAGTTCCAACCTGAACC | 60 |
| 5800 | ATCCCAGGTCCCAATTTTCTTC | CCATACCTGAGGGACCTGAAAC | 57 |
